# A Graph Neural Network Based Decentralized Learning Scheme

**DOI:** 10.3390/s22031030

**Published:** 2022-01-28

**Authors:** Huiguo Gao, Mengyuan Lee, Guanding Yu, Zhaolin Zhou

**Affiliations:** 1 College of Information Science and Electronic Engineering, Zhejiang University, Hangzhou 310027, China; huiguogao@zju.edu.cn (H.G.); mengyuan_lee@zju.edu.cn (M.L.); 2 Zhejiang Provincial Key Laboratory of Information Processing, Communication and Networking (IPCAN), Hangzhou 310027, China; 3 College of Control Science and Engineering, Zhejiang University, Hangzhou 310027, China; 3170103159@zju.edu.cn

**Keywords:** decentralized learning, graph neural network, average consensus

## Abstract

As an emerging paradigm considering data privacy and transmission efficiency, decentralized learning aims to acquire a global model using the training data distributed over many user devices. It is a challenging problem since link loss, partial device participation, and non-independent and identically distributed (non-iid) data distribution would all deteriorate the performance of decentralized learning algorithms. Existing work may restrict to linear models or show poor performance over non-iid data. Therefore, in this paper, we propose a decentralized learning scheme based on distributed parallel stochastic gradient descent (DPSGD) and graph neural network (GNN) to deal with the above challenges. Specifically, each user device participating in the learning task utilizes local training data to compute local stochastic gradients and updates its own local model. Then, each device utilizes the GNN model and exchanges the model parameters with its neighbors to reach the average of resultant global models. The iteration repeats until the algorithm converges. Extensive simulation results over both iid and non-iid data validate the algorithm’s convergence to near optimal results and robustness to both link loss and partial device participation.

## 1. Introduction

With the immense growth of data and the exponential increase in computation power, great attention has been given to the machine learning techniques, which has superior performance for classification, regression, anomaly detection, denoising, and translation tasks. However, the issue of long runtime for training the models on a single machine becomes the main bottleneck for large-scale applications. This motivates us to use distributed systems because of their increasing parallel computation power.

### 1.1. Literature Review

There are two ways to realize the distributed machine learning, centralized and decentralized. For the centralized way, there is always a central coordinator to help distributed devices collaboratively train a machine learning model. For example, each device with the local dataset holds the initial model and computes local gradients in centralized parallel stochastic gradient descent (CPSGD) algorithms [1,2,3]. It then uploads the gradient to the central server. The central server aggregates the gradients and sends the average value to each device. Finally, each device performs updates based on the received gradients. This method achieves the linear speedup compared to operating on a single machine. Recently, some popular centralized learning frameworks [4,5] are proposed, which further take the security into consideration.

However, the above scheme requires all devices in the network to communicate with the central server concurrently, which would induce severe traffic jam. To overcome this defect, decentralized learning aims to obtain a global machine learning model by minimizing the summation of local loss functions without the central coordinator. In decentralized learning, each device needs to exchange information with their neighbors, which avoids possible traffic jam especially on networks with limited bandwidth. Recently, several decentralized algorithms have been proposed in the literature, and we summarize them in Table 1. Among them, distributed gradient descent (DGD) [6,7,8] is one of the most efficient algorithms. When the loss function is strongly convex, the algorithm has the sublinear convergence rate to the optimal results with diminishing step sizes. However, if the algorithm adopts the constant step size, it cannot always converge to the optimal results. To guarantee convergence to the optimum with constant step sizes, the authors in [9] utilize the gradient tracking technique and propose a decentralized exact first-order algorithm (EXTRA). The DIGing algorithm in [10] also utilizes the gradient tracking techniques but considers more complex situations like the variant network scenario as well as both directed and undirected graphs. When the graph is time-invariant and undirected, the algorithm is equivalent to the EXTRA algorithm. For the decomposition techniques like decentralized ADMM (DADMM), they decompose the initial problem into several sub-problems and updates the optimization variables by solving the sub-problems step by step. The DADMM algorithm establishes its linear convergence for strongly convex local objectives [11]. However, due to the fixed update rule, DADMM cannot flexibly accommodate the communication-computation tradeoff and it requires additional hyperparameters to tune. The authors in [12] propose COLA based on its centralized form, CoCoA, to overcome these drawbacks but it can only be applied to linear models. Moreover, it guarantees linear convergence rates for strongly convex loss functions and sublinear convergence rates for convex loss functions.

However, the above-mentioned algorithms based on full gradient descents such as DGD, EXTRA, and DIGing generally consume unbearable time. In addition, some algorithms like DADMM rely on the exact solution of subproblems or fine-tuning of hyperparameters, and others like COLA have limits on the model type. To overcome the above shortcomings, we focus on the parallel SGD scheme that can efficiently train most machine learning models based on the stochastic gradient. There are many distributed parallel SGD (DPSGD) algorithms in which each user device computes stochastic gradients locally and averages model parameters with its neighbors. Some [13] assumed that user devices in the network could perform computation and communication at the same time but some assumed not [14,15,16,17]. If considering a device can compute and communicate at the same time, one must take into account resource allocation for computation and communication of the device. In this paper, for simplicity, we consider the algorithm in which each device cannot perform computation and communication simultaneously.

### 1.2. Main Contribution

The main problem of DPSGD lies in that it can only aggregate the model information in the neighborhood instead of the global network. Under the non-iid setting, it would suffer from long iteration time when the training data features of neighborhood cannot reflect the data features of global network. Thus, we should average all model parameters in the global network, which is known as average consensus in literature [18]. Meanwhile, graph neural networks (GNNs) have become the research hotspot recently and have been utilized in the communication area to achieve better machine learning performance [19,20,21]. They are novel neural networks for processing graph data over the non-Euclidean space. GNN is motivated by convolutional neural network (CNN) and graph embedding. To extend the generalization of CNNs to graphs, GNNs are proposed to aggregate information from generalized graph structures and learn the embedding vector of each node as output by exchanging information with neighbors in a decentralized manner. As for the considered decentralized learning system, the network composed of user devices can be modeled as a graph. Since GNNs have shown superior performance on graph data and they can be implemented in a decentralized manner, it is natural to combine GNNs with decentralized learning. Besides, GNNs are scalable to the graph size and topology, which show great scalability to unseen graphs and are robust to the dynamic distributed environment. Therefore, in this paper, we propose to use GNN to replace the original model aggregation in DPSGD, which is the most intractable part in the DPSGD. In this way, we develop a new decentralized learning scheme. Specifically, a GNN model is first trained for average consensus and kept at each device. After that, each device updates local models based on local datasets and exchanges information with each other. Then, each device uses the trained GNN models and its neighbor’s model information to get the global average model. It avoids the congestion to the central server since each device only communicates with their neighbors. The process repeats until the algorithm converges. Our main contribution in this paper is to propose a new decentralized learning scheme. It uses GNN aggregation for training generalized models in networks that can be modeled as undirected or balanced directed graphs. The novelty of our algorithm lies in that we utilize the GNNs in decentralized learning scheme and it achieves excellent performance compared against various existing methods. Specifically, our algorithm offers:

- *Convergence:* The simulation results show that the proposed algorithm can converge to near-optimum under both iid and non-iid setting. Particularly, our algorithm outperforms DPSGD on the time-invariant topology under the non-iid setting.
- *Computation Efficiency:* By averaging its model within global connected network, each user device reduces computational costs when achieving the same performance as DPSGD under the non-iid setting. By exploiting decentralized learning using SGD, the proposed algorithm ensures fast convergence compared with full gradient descent based algorithms.
- *Robustness:* We experimentally validate that the proposed algorithm is resilient to link loss and partial device participation.

### 1.3. Organization

We organize the rest of paper as follows. We introduce the system model and the decentralized learning tasks in Section 2. Next, we present the details of GNN aggregation based decentralized learning scheme in Section 3. Since GNN aggregation is used in our decentralized learning scheme and would affect its performance, we verify the effectiveness of GNN aggregation that aims to reach average consensus on random geometric graphs (RGGs) [22] in Section 4. Then, we show the performance of the proposed algorithm where GNN aggregation is embedded in Section 5. Finally, we conclude the whole paper in Section 6. The abbreviations used in the article are listed in Table 2.

## 2. Decentralized Learning Tasks over Network

### 2.1. System Model

**Figure 1:**
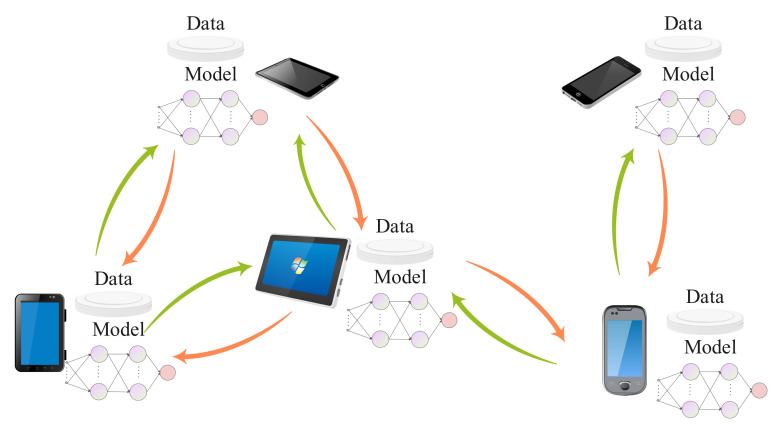
The system model.

We consider a wireless system where *K* mobile devices collaboratively train a machine learning model denoted as w to support a specific application, e.g., image classification or natural language processing, as shown in Figure 1. We denote the mobile devices as the set K={1,2,…,K} and each device *k* holds a set of training data denoted as $D_{k}=\left\{\left(x_{k}^{1},y_{k}^{1}\right),\left(x_{k}^{2},y_{k}^{2}\right),\ldots ,\left(x_{k}^{n_{k}},y_{k}^{n_{k}}\right)\right\}$, where $x_{k}^{i}$ means the *i*-th training data sample, $y_{k}^{i}$ represents the corresponding ground-truth label, and $n_{k}$ is the size of the training set $D_{k}$. We let $n=\sum_{k=1}^{K}n_{k}$ denote the total number of training samples over all devices in the network. The global machine learning model $w\in R^{D}$ is trained over all the distributed data and each device has its own current model. Then, the total model parameters in the system can be formulated as

(1) $$W≜{\left[w_{1},w_{2},\ldots ,w_{K}\right]}^{⊤}≜\left[w^{1},w^{2},\ldots ,w^{D}\right]\in R^{K\times D},$$

where $w_{k}\in R^{D}$, each row of W, represents the model parameters of device *k* and $w^{d}\in R^{K}$, each column of W, corresponds to all devices’ model parameters at the dimension *d*. To protect the data privacy, devices exchange their model information instead of original training data over the wireless channel. We assume that each device can communicate with others within certain distance and the transmission is perfect without any errors in received signals. This can be guaranteed by robust channel coding techniques, which has been assumed in many other literatures, such as [23,24]. All the devices in the network proceed a synchronous update scheme. For machine learning operating on a single node, model training is to minimize the loss function on all the local training data. Therefore, the overall training objective for decentralized learning can be formulated as

(2) $$\min_{w\in R^{D}}f\left(w\right)=\frac{1}{n}\sum_{k=1}^{K}n_{k}F_{k}\left(w\right),$$

where $F_{k}\left(w\right)=\frac{1}{n_{k}}\sum_{i=1}^{n_{k}}f_{i}\left(w,x_{k}^{i},y_{k}^{i}\right)$ denotes the local loss function of device *k* on its local dataset $D^{k}$. Here, $f_{i}\left(w,x_{k}^{i},y_{k}^{i}\right)$ is the sample-wise loss function on sample $\left(x_{k}^{i},y_{k}^{i}\right)$ with model parameter w. If the training samples are distributed over the user devices uniformly at random, the expectation over the set of examples is equal to f(w), i.e, $E(F_{k}\left(w\right))=f\left(w\right)$,∀k and this is the independent and identically distributed (iid) assumption. Then, Problem (2) can be formulated into

(3) $$\min_{w\in R^{D}}f\left(w\right)=\frac{1}{K}\sum_{k=1}^{K}F_{k}\left(w\right).$$

If the equation does not hold, we refer to it as the non-iid setting.

### 2.2. Decentralized Learning Model

Problem (2) is a typical decentralized learning problem. There are many decentralized learning algorithms that have been proposed as described in Section 1 to solve this problem. Here, we focus on the parallel SGD scheme where devices perform SGD locally and then aggregate the model parameters. It can efficiently handle most machine learning models based on stochastic gradients. Due to the absence of central coordinator, DPSGD can only aggregate the model information in the neighborhood instead of the global network. Thus, we want to do average consensus across the network to help devices acquire the global information at the absence of central coordinator. In the network formed by the agents, “consensus” means that all agents reach the same state. The “consensus algorithm” can be correspondingly defined as the interaction rule of exchanging information between nodes and their neighbors [18]. The consensus problem is very important in coordination tasks of robots or unmanned aerial vehicle (UAV), information fusion in sensor networks, and federated learning [25]. Among numerous linear or nonlinear consensus problems, distributed averaging consensus may be the most common consensus problem over a network. The goal of average consensus is to let each node reach the same average value of all nodes. It can be done in many ways including flooding, distributed linear iterations [26], deep neural networks [27], graph filters [28], and graph convolutional neural networks [29]. In this paper, we leverage the GNN [29] to reach average consensus on global model parameters for the following reasons. First, as for the decentralized learning setting, the network composed of devices can be modeled as a graph with the set of nodes and each link between devices can be seen as an edge. As mentioned before, GNNs have great potential for processing graph data and therefore is suitable to the system. Next, GNNs can be implemented in a decentralized way, which fits the decentralized setting with no central coordinator. Moreover, GNNs are scalable to the size of graph, that is, well-trained GNNs can take the graph with any number of nodes as input which adapts the dynamic distributed environment. Last but not least, GNNs do not need high-precision matrix decomposition to get the parameters, as compared to the aforementioned graph filters [28]. They have shown great scalability to unseen graphs and are robust to the link loss [29].

Specifically, our proposed algorithm is summarized in Algorithm 1. At the beginning of iteration *k*, each node *i* samples batches of data instances $\xi_{k,i}$ from its local dataset randomly. Then, node *i* computes the local stochastic gradient $\nabla F_{i}\left(x_{k,i};\xi_{k,i}\right)$ and updates local models with the diminishing step size $\alpha_{k}$. The step size at the *k*-th iteration $\alpha_{k}$ is obtained based on the given step size α considering the mean and variance of the past gradients [30]. Finally, each node utilizes a well-trained GNN model and exchanges information with their neighbors to reach the average of resultant global models. The iteration repeats until the algorithm converges. In the following section, we will introduce how to train a GNN model for the weight aggregation, i.e., Step 7 in Algorithm 1.
**Algorithm 1** A GNN Based Decentralized Learning Scheme1:**Input:** initial $x_{0,i}=x_{0}$, step size α, trained graph neural network, eigenvalue λ and the number of iterations *K*.2:**for** k=0,1,2,…,K−1 **do**3: Sample $\xi_{k,i}$ from the local dataset of the *i*-th node randomly.4: Compute the local stochastic gradient $\nabla F_{i}\left(x_{k,i};\xi_{k,i}\right)$, ∀i for all nodes.5: Compute $\alpha_{k}$ based on α, the mean and variance of the past gradients.6: Update the local optimization variable $x_{k+\frac{1}{2},i}\leftarrow x_{k,i}-\alpha_{k}\nabla F_{i}\left(x_{k,i};\xi_{k,i}\right)$.7: Compute the average optimization variables $x_{k+1,i}=\frac{1}{N}\sum_{i=0}^{N-1}x_{k+\frac{1}{2},i}$ using trained GNN’s weights and λ (see **Algorithm 2**).8:**end for**

## 3. Gnn Aggregation Based Average Consensus

As mentioned in Section 2, we want to embed GNN aggregation into the parallel SGD scheme to develop a new decentralized learning scheme. In this section, we begin with the introduction to the GNN structure. Then, we model the network to a graph and explain the details of the GNN structure to reach average consensus. Moreover, we illustrate its training process and finally illustrate the decentralized GNN aggregation.

### 3.1. Overview of GNN

We consider a graph G(V,E), where V is the set of *N* nodes, i.e., |V|=N and E is the set of *M* edges, i.e., |E|=M. GNN aims to learn a state embedding $h_{v}\in R^{s}$, an *s*-dimension vector, for each node *v*. Then $h_{v}\in R^{s}$ is utilized to produce an output $o_{v}$ for each node. To achieve the above goals, GNNs take node features, edge information, and matrix representations of the graph as input. GNNs are often hierarchical models with multiple layers. In each layer, each node shares the same transition function *f* and the same output function *g*. Given *f* and *g*, $h_{v}\in R^{s}$ and $o_{v}$ at the *l*-th layer can be defined as

(4) $$h_{v}^{l}=f^{l}\left(x_{v},x_{co[v]},h_{ne[v]}^{l-1},x_{ne[v]}\right),$$

(5) $$o_{v}^{l}=g^{l}\left(h_{v}^{l},x_{v}\right),$$

where $x_{v}$, $x_{co[v]}$, $x_{ne[v]}$ are the features of node *v*, edges connected to node *v*, and node *v*’s neighbors, respectively; $h_{ne[v]}^{l-1}$ is the embeddings held by neighbors of node *v* at the (l−1)-th layer. From the above equations, we can conclude that each node only needs to utilize its own features and its neighbors’ information to get the predicted output, which explains why GNN can be implemented in a decentralized way.

### 3.2. Gnn for Consensus

**Figure 2:**
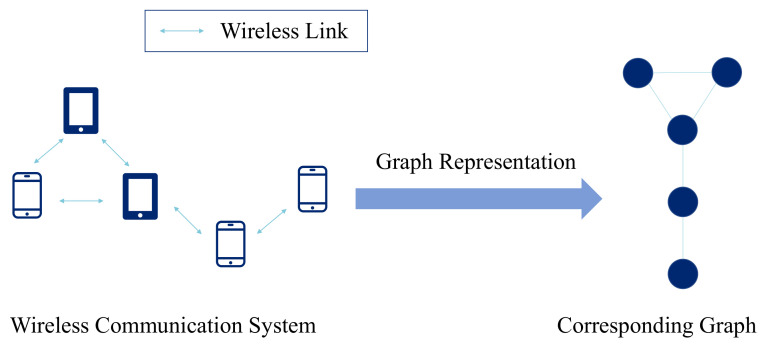
Graph representation for the considered wireless communication system shown in Figure 1.

To utilize GNN for consensus, we first need to explore the graph representation for the wireless system in Figure 1. In the aforementioned communication network, each user device can be seen as a node and each link can be seen as an undirected edge. Two nodes are connected by an edge if they can communicate with each other. Each device’s model parameters can be seen as its features. We denote the feature of node *i* as $w_{i}=\left[w_{i}^{1},w_{i}^{2},\ldots ,w_{i}^{D}\right]\in R^{D}$. The corresponding graph representation is shown in Figure 2.

However, we cannot take $w_{i}$ for node *i* as the input graph signals directly since it would lead to an oversized GNN model and therefore bring difficulties to training when the number of features *D* increases. As we can compute the consensus version of features for each dimension successively, we consider that node *i* chooses $w_{i}^{d}$ as its input graph signal, where $w_{i}^{d}$ denotes the initial node features at the dimension *d*. We denote all nodes’ features at the dimension *d* as $w^{d}=\left[w_{1}^{d},w_{2}^{d},\ldots ,w_{N}^{d}\right]\in R^{N}$. For the whole graph, we consider $w^{d}\in R^{N}$ as the input graph signal and the consensus version ${\bar{w}}^{d}={\bar{w}}^{d}1$ as the desired output, where ${\bar{w}}^{d}$ denotes the average of input features at dimension *d* and 1 represents the vector whose all coefficients are 1. Here, consensus can be regarded as applying GNN to transfer the input graph signal into constant output graph signals. We consider a GNN composed of *L* graph convolutional layers in series with a fully-connected layer. The graph convolution is defined as a linear-and-sum operation [31]. Given a set of parameters $\theta ={\left[\theta_{0},\ldots ,\theta_{K}\right]}^{⊤}$, the graph convolution is formulated as

(6) $$\Theta \left(S\right)w_{in}=\sum_{k=0}^{K}\theta_{k}S^{k}w_{in},$$

where S denotes the graph shift operator (GSO) matrix and $w_{in}$ denotes the input graph signals. The GSO matrix indicates the connection between nodes. For each entry ${\left[S\right]}_{ij}=s_{ij}$, we have $s_{ij}\neq 0$, if (i,j)∈E or i=j. We can use the adjacency matrix A, the Laplacian matrix L, and their normalized or translated forms to represent S. Here, we utilize the normalized form of the adjacency matrix as the GSO matrix, i.e., $S=A/\lambda_{\max}\left(A\right)$. The graph convolution filters the input graph signal $w_{in}$ with an FIR graph filter Θ(S). We will compare the performance of FIR graph filter and GNN later in Section 4. As for GNN, each graph convolutional layer consists of several graph filters and a nonlinear function. At the *l*-th convolutional layer, the GNN takes $F_{l-1}$ input features ${\left\{w_{l-1}^{g}\right\}}_{g=1}^{F_{l-1}}$ and produces $F_{l}$ output features ${\left\{w_{l}^{f}\right\}}_{f=1}^{F_{l}}$. Each input feature $w_{l-1}^{g}$ is considered as a graph signal and processed by a bunch of graph filters ${\left\{\Theta_{l}^{fg}\right\}}_{f}$. The filter outputs are summarized over the input index *g* to produce the aggregated *f*-th intermediate feature

(7) $$z_{l}^{f}=\sum_{g=1}^{F_{l}}\Theta_{l}^{fg}\left(S\right)w_{l-1}^{g}=\sum_{g=1}^{F}\sum_{k=0}^{K}\theta_{kl}^{fg}S^{k}w_{l-1}^{g}.$$

Then it passes the activation function σ(·) and outputs the *f*-th feature of the *l*-th layer, as

(8) $$w_{l}^{f}=\sigma \left(z_{l}^{f}\right),$$

where the activation function σ(·) is taken as the ReLU function in this paper. Equations (7) and (8) denote the process of each node generating a state embedding as shown in (4). After passing through all *L* convolutional layers, we denote the output of the *L*-th convolutional layer as $w_{L}=\left[w_{L}^{1},w_{L}^{2},\ldots ,w_{L}^{F_{L}}\right]\in R^{N\times F_{L}}$. Then, the *L*-th layer convolutional features are passing through the fully-connected layer to get the final output

(9) $$w_{\mathrm{out}}=w_{L}\theta_{FC},$$

where $\theta_{FC}={\left[\theta_{FC}^{1},\theta_{FC}^{2},\ldots ,\theta_{FC}^{F_{L}}\right]}^{T}$ is the $F_{L}\times 1$ vector of the fully-connected layer. Here, Equation (9) denotes the process of each node generating the final output as shown in (5). In this paper, we consider the number of convolutional layers L=2 and the number of features $F_{1}=F_{2}=F=32$.

### 3.3. Training Process

We sample the data generated by running CPSGD algorithms and calculate the average output to form a training pair. The reasons are as follows. First, it is hard to generate the same distribution of training data as the data needed to aggregate when implementation, while the data generated by running CPSGD algorithms share similar distribution. In addition, considering the situation that decentralized learning serves as the substitute of centralized learning, it is natural to utilize the data generated by the latter to train the former and the data are easy to obtain. The communication cost during the training process is mainly caused by the model update depending on different wireless scenarios [32,33]. Then we use the mean squared error (MSE) loss as the loss function to train the GNN in a supervised way. Depending on the sampling location of data, sampling strategies can be divided into three categories.

- *Head-sampling*: Sample the data from the first 10% epochs by running CPSGD.
- *Tail-sampling*: Sample the data from the end 10% epochs by running CPSGD.
- *Uniform-sampling*: Sample the data at equivalent round intervals and the total number of data is equal to 10%.

We will verify the performance of three different sampling strategies in Section 5.

### 3.4. Decentralized GNN Aggregation

**Figure 3:**
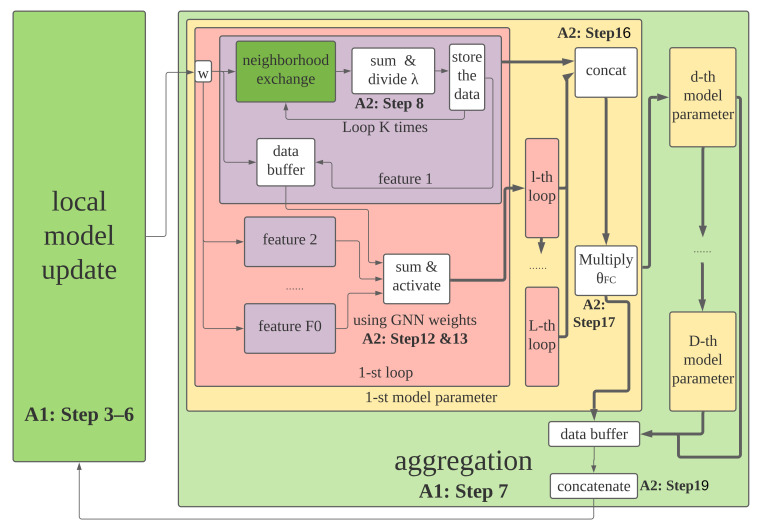
The architecture of proposed decentralized learning scheme.

With the above training process, a GNN aggregation model can be trained and kept in each device. In this part, we introduce how to implement the well-trained GNN aggregation in a decentralized manner. The detailed process is summarized as Algorithm 2. Specifically, after obtaining the well-trained GNN model and the max eigenvalue λ of the current typology’s adjacency matrix that can also be acquired by decentralized algorithms [34], we distribute the GNN’s all parameters and λ to each node. Take node *i* as an example. It gets all convolution coefficients $\theta_{k,l}^{f,g}$, ∀k∈K, ∀l∈L, $\forall f\in F_{l}$, $\forall g\in F_{l-1}$, from *L* convolutional layers and $\theta_{FC}$ from the fully connected layer. Here, $\theta_{k,l}^{f,g}$ means the weight over the input index *g* to the *f*-th intermediate feature for the *k*-th recursion of the *l*-th convolutional layer. In Algorithm 2, $w_{i,l}^{f,d}$ means the *f*-th feature of node *i* at the *l*-th convolutional layer to get the initial feature at dimension *d*. Then, each node utilizes these parameters to iterate based on both models of its own and its neighbors. Remind that there is a synchronous protocol to guarantee all nodes proceed the same loop. Finally, each node acquires the average of resultant models in the whole network. To better demonstrate the architecture of the proposed decentralized learning scheme, we use the graph representation as shown in Figure 3. The communication and computation complexities of the proposed decentralized learning scheme are O(MK) and $O\left(F^{2}LMK\right)+O\left(NF\right)+O\left(D/\epsilon \right)$, respectively, where ϵ is the error measured by loss functions.
**Algorithm 2** Decentralized GNN Aggregation on RGG1:**Input:** the dimension of node’s initial feature *D*, the number of convolutional layers *L*, filter order *K*, the number of features of the *l*-th convolutional layer $F_{l}$ ($F_{0}$ is the input dimension), the input feature of each node $w_{i}$, weight $\theta_{k,l}^{f,g}$ of convolutional layers, weight vector $\theta_{FC}$ of the fully connected layer, eigenvalue λ.2:**for all** nodes i∈N in parallel **do**3:  **for** d=1 to *D* **do**4:   **for** l=1 to *L* **do**5:     **for** f=1 to $F_{l-1}$ **do**6:        $t_{i,l}^{f,0}=w_{i,l-1}^{f,d}$7:        **for** k=1 to *K* **do**8:          $t_{i,l}^{f,k}=\sum_{j\in N_{i}}\frac{1}{\lambda }t_{j,l}^{f,k-1}$9:        **end for**10:     **end for**11:     **for** f=1 to $F_{l}$ **do**12:        $z_{i,l}^{f}=\sum_{k=0}^{K}\sum_{g=1}^{F_{l-1}}\theta_{k,l}^{f,g}t_{i,l}^{g,k}$13:        $w_{i,l}^{f,d}=\sigma \left(z_{i,l}^{f}\right)$14:     **end for**15:   **end for**16:   $w_{i,L}^{d}:=\left[w_{i,L}^{1,d},w_{i,L}^{2,d},\ldots ,w_{i,L}^{F_{L},d}\right]$17:   $w_{i}^{d}=w_{i,L}^{d}\theta_{FC}$18:  **end for**19:  
$w_{i}:=\left[w_{i}^{1},w_{i}^{2},\ldots ,w_{i}^{D}\right]$20:**end for**

## 4. Performance Evaluation on Average Consensus

In this section, we show simulation results to test the performance of GNN based average consensus on RGGs and validate the performance advantages compared with the FIR graph filters.

### 4.1. Simulation Settings

The simulation settings are given as follows. We employ the same GNN structure as [29], which is composed of two graph convolution layers and a fully connected layer. Specifically, we choose the normalized form of the adjacency matrix as the GSO matrix S in Equation (7) and vary the filter order to see the performance difference. We set each graph convolution layer’s feature as 32 and choose the activation function as ReLU function. Besides, the fully connected layer has 32 units. As for the dataset, we use our generated data to train and test the models. First, we generate the graph samples, which contains 2500 training samples, 250 validation samples and 250 test samples. To generate each graph sample, we distribute 100 nodes randomly in a square area with side 100 m and connect two nodes with an undirected edge if their distance is smaller than 20 m. Then, we generate each node’s feature from the standard normal distribution and calculate their average value as the label for each graph sample. After that, we process the test set by randomly removing the edge or node with some certain probabilities. As for the training parameters, we choose Adam optimizer with all default parameters recommended by [30], set the batch size as 100 and run 400 epochs to minimize the MSELoss between the output and the target value. Finally, we get the model that performs the best on the validation set and test the performance on the processed test set. For comparison, the FIR graph filter used for average consensus is also implemented.

### 4.2. Performance of GNN with Different Filter Orders

**Figure 4:**
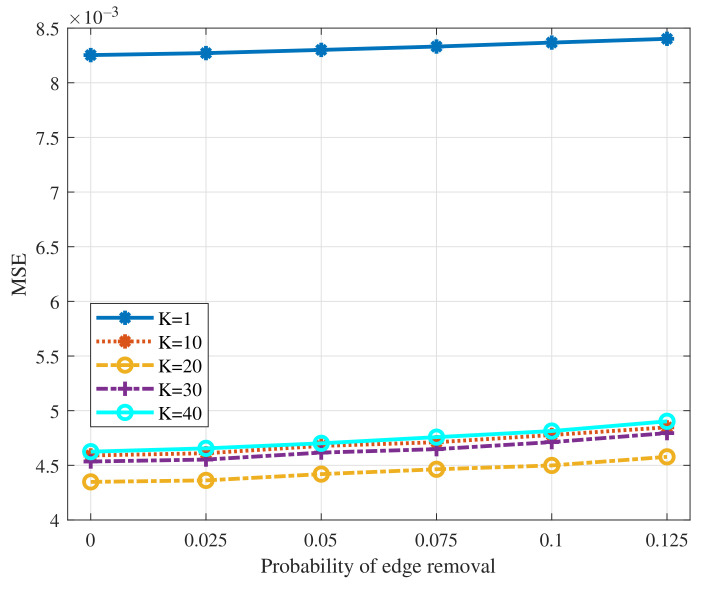
MSELoss of GNN to the edge removal with different filter orders.

**Figure 5:**
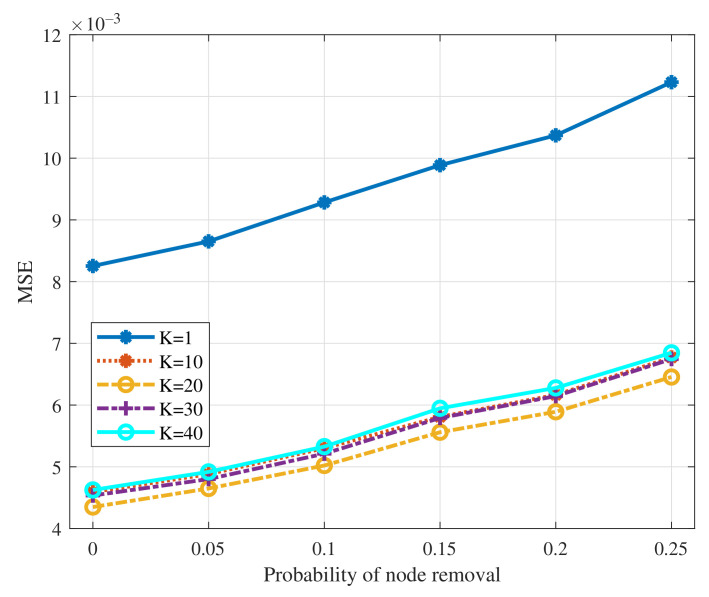
MSELoss of GNN to the node removal with different filter orders.

We test the performance of GNN with the filter order varying in [1,10,20,30,40] and the probability of edge removal varying within the interval [0,0.125]. The results are summarized in Figure 4. From the figure, we can see that the MSELoss of GNN increases slightly as the probability of edge removal increases. It shows the robustness of GNN doing average consensus when some nodes lose the connection with their neighbors, since GNN has learned to capture the structure of graph to reach average consensus through the training process. Besides, when the probability of edge removal is fixed, the MSELoss decreases first and then increases as the filter order increases. The reasons are as follows. The average consensus can be regarded as a low-pass filter in the frequency domain while we need high orders to design such a perfect low-pass filter. Models’ performance would be degraded significantly under the low filter order setting no matter how the limited parameters change. From the perspective of message passing, the filter order *K* indicates that each node can utilize at most *K*-hop neighbors’ information. It is difficult for each node to get the exact global average value over large sparse graphs while the filter order is relatively small. On the other hand, when the filter order increases, the tunable parameters increase but it cannot always guarantee the better performance since more difficulties would be brought at the same time. Note that when the filter order is 20, GNN performs the best since it is a proper value regarding the graph size. We then test the performance of GNN with the filter order varying in [1,10,20,30,40] as the probability of node removal varies within the interval [0,0.25]. The results are summarized in Figure 5. A similar result as the scenario of the edge removal can be observed from the figure, that is, the MSELoss increases slightly as the probability of node removal increases. Note that we do not need to re-train a new GNN when the input number of nodes changes since the operation in the GNN is node-invariant.

### 4.3. Performance Comparison with FIR Graph Filters

**Figure 6:**
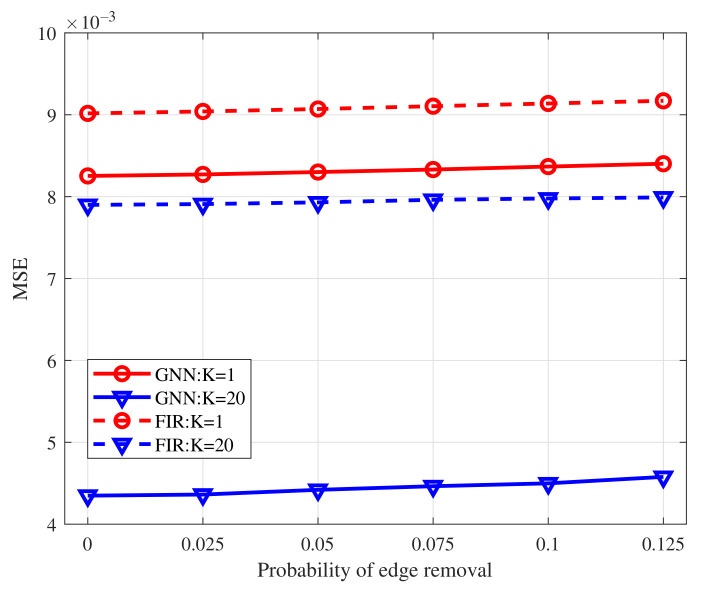
MSELoss of GNN to the edge removal compared with FIR graph filters.

**Figure 7:**
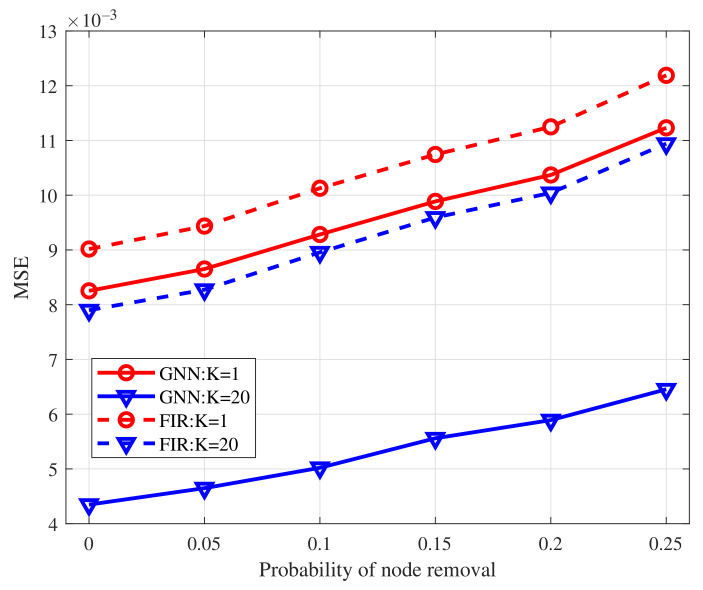
MSELoss of GNN to the node removal compared with FIR graph filters.

We compare the MSELoss of GNN and FIR graph filters with filter order K=1 and K=20 as the probability of edge removal varies within the interval [0,0.125]. The results are shown in Figure 6. From the figure, the GNN outperforms the FIR graph filters for the same filter order. Specifically, given the filter order K=20, the MSELoss achieved by GNN is nearly half of that achieved by FIR graph filters. The performance improvement of GNN, with regard to FIR graph filters comes from the added nonlinear function and multi-layer structure. Empowered by them, the GNN can capture more features from the graph structure and therefore achieves lower MSELoss when doing average consensus than FIR graph filters. We then compare the MSELoss of GNN and FIR graph filters with K=1 and K=20 as the node removal probability changes within the interval [0,0.25]. The results are shown in Figure 7. From the figure, the errors of both GNN and FIR graph filters are enlarged with the increase of node removal probability. It is because that removing more nodes would cause greater changes to the initial graph topologies. As the number of removal nodes increases, more and more isolated nodes appear, which cannot exchange information with neighbors and therefore achieves higher MSELoss. Owing to the nonlinear operation and multi-layer architecture, GNN has a smaller error increase than FIR graph filters. Besides, GNN achieves lower MSELoss than the FIR graph filters with the same filter order. The results demonstrate that GNN is scalable to different topologies.

## 5. Performance Evaluation over Decentralized Learning

In Section 4, we have verified the effectiveness of GNN doing average consensus on RGGs and its scalability to different topologies. In this section, we then test the performance of the proposed decentralized learning scheme in which GNN based average consensus is embedded.

### 5.1. Simulation Settings

The simulation settings are given as follows. We consider a wireless communication system consisting of N=100 devices. The devices are randomly distributed in a square area with side 100 m and the link used for data transmission is established if the distance between two devices is smaller than 20 m. We generate graph samples to model the connection between devices, which contains 2000 training samples, 250 validation samples, and 250 test samples. In terms of the decentralized learning model, we choose the multiple linear regression models added a softmax function. We then choose two datasets to test our algorithm. One of them is the popular MNIST dataset, which is composed of 60,000 training samples and 10,000 test samples with 10 classes. As for the other one, we choose more complex Fashion-MNIST [35] dataset, where the image size, the number of training and test samples are the same as MNIST. Since we here consider the mobile data to be iid, we randomly divide the training samples into *N* equal parts and distribute them to all devices, respectively. Before performing the decentralized learning tasks, we must train the GNN first over the training graph samples. The GNN structure is the same as that in Section 4. For the training process, it has already been described in Section 3, where the epoch of running CPSGD algorithm is set as 250. Then, we can use the well-trained GNN models to aggregate the decentralized learning model parameters in the decentralized learning tasks as described in Section 3. For comparison, CPSGD and DPSGD algorithms are also implemented in the test. We run each algorithm for 250 epochs and average its performance over 250 test graph samples.

### 5.2. Performance Comparison with CPSGD and DPSGD

Table 3 shows the average test accuracy of the proposed algorithm as well as the baseline algorithms. From the table, we can see that performance of the proposed algorithm is very close to that of CPSGD and DPSGD. Specifically, the proposed algorithm with the head-sampling strategy achieves 2.50% lower accuracy than CPSGD when filter order K=1 and 2.04% lower accuracy when filter order K=20 for the MNIST dataset. Meanwhile, for the Fashion-MNIST dataset, it achieves 7.65% lower accuracy when K=1 and 5.85% lower accuracy when K=20. The accuracy gap comes from the error of GNN doing average consensus. Since the distribution of data needed to aggregate is slightly different from the training data, trained GNN would have a slightly worse performance than the optimum. However, errors of GNN doing average consensus would not be accumulated due to the model update step in Algorithm 1 and therefore the proposed algorithm can still converge to the near-optimal results. Compared with DPSGD, the proposed algorithm with the head-sampling strategy achieves 2.47% lower accuracy when filter order K=1 and 2.01% lower accuracy when K=20 for the MNIST dataset. Meanwhile, for the Fashion-MNIST dataset, it achieves 7.18% lower accuracy when K=1 and 5.38% lower accuracy when K=20. The reasons are as follows. Since the features of mobile data in the neighborhood are the same with those in the global network under the iid setting, the advantage of aggregating model parameters across the network is not more obvious than aggregating in the neighborhood.

### 5.3. Performance Comparison between Different Sampling Strategies

From Table 3, we can find that the proposed algorithm with the head-sampling strategy performs the best among all three sampling strategies. Specifically, the proposed algorithm with the head-sampling strategy achieves 0.86% higher accuracy than uniform-sampling and 1.58% higher accuracy than tail-sampling when K=1, 0.89% higher accuracy than uniform-sampling and 1.18% higher accuracy than tail-sampling when K=20 for the MNIST dataset. Meanwhile, for the Fashion-MNIST dataset, it achieves 0.32% higher accuracy than uniform-sampling and 0.41% higher accuracy than tail-sampling when K=1, 0.17% and 1.22% higher accuracy than uniform-sampling and tail-sampling, respectively, when K=20. It is because that head-sampling strategy makes the proposed algorithm exploit more training data with considerable variation, which benefits the practical implementation. As a reminder, we collect the machine learning model parameters from each round of the CPSGD algorithm as training data. For the beginning rounds, model parameters on each device are randomly generated and there is a tremendous difference from model to model. As the training process continues, the model on each device becomes more and more similar. Finally, the model on each device becomes almost the same and the CPSGD algorithm converges. Thus, the training data sampled from the beginning rounds are more diverse than those from the end rounds, which prevents the GNN from over-fitted training and help it learn a more accurate model.

### 5.4. Scalability to Scenarios with Different Topologies

**Figure 8:**
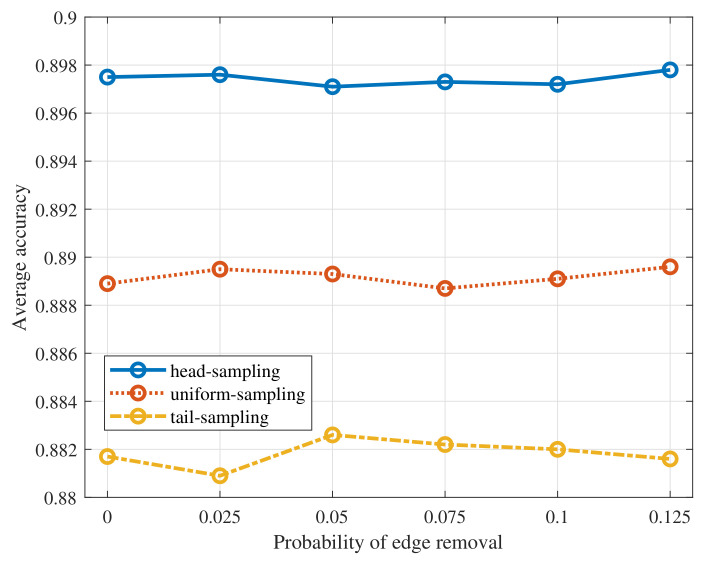
Average accuracy of the proposed algorithm to the edge removal.

**Figure 9:**
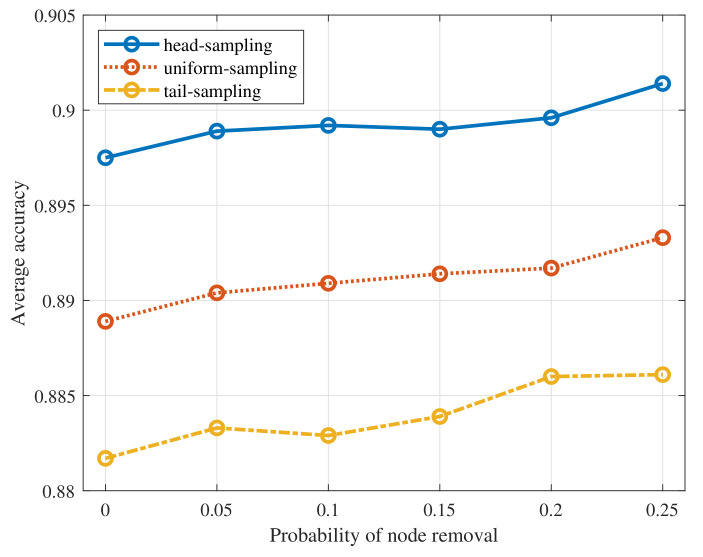
Average accuracy of the proposed algorithm to the node removal.

To simulate the network being affected by the link loss, we randomly remove the edge of test graph samples as the probability varies within the interval [0,0.125]. Then, we test the performance of the proposed algorithm with three sampling strategies when the filter order K=1 on the MNIST dataset. The results are summarized in Figure 8. From the figure, we can find that the average accuracy of the proposed algorithm remains almost unchanged as the probability of link loss increases. The reasons are as follows. First, GNN based average consensus is robust to the edge removal as we have validated in Section 4. Thus, using GNN to aggregate the model parameters across the network can still obtain the accurate averaged global model parameters. Besides, with link loss, errors of GNN reaching average consensus increase but the model update step in Algorithm 1 would counteract its negative effects and therefore improves the stability of the algorithm. To simulate the situation that not all devices participate in the training process, we randomly remove the node of test graph samples with the probability varying within the interval [0,0.25]. Then, we test the performance of the proposed algorithm with three sampling strategies when K=1. The results are shown in Figure 9. From the figure, the average test accuracy remains almost unchanged. It is because that utilizing GNN to reach average consensus is also robust to the node removal as we have verified in Section 4. Moreover, the model update step in Algorithm 1 enhances the robustness of the proposed algorithm.

### 5.5. Non-Iid Scenario

**Figure 10:**
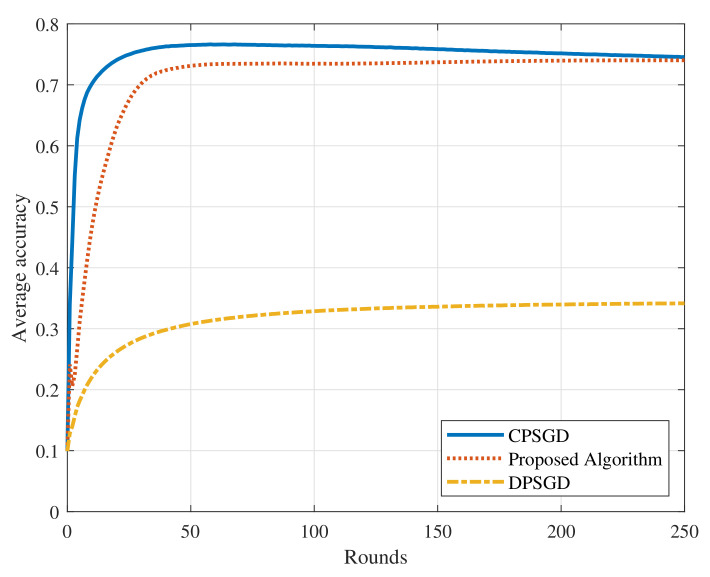
Comparison with other methods on the non-iid dataset.

Since the mobile data distribution is affected by many factors including geographic position, users’ different preferences, the interaction between individuals and social groups, and so on, we consider the more general non-iid data partition way in this part. The training samples from the MNIST dataset are sorted according to their classes first. We then divide them into *N* equal parts, and distribute them to all devices, respectively. For simplicity, we fix the graph topology and then gather the training data using the head-sampling strategy as described in Section 3. As for the GNN structure, we choose the filter order K=20 to enhance the ability of GNN doing average consensus. The other simulation settings are the same as the iid scenario. We then test the proposed algorithm and choose CPSGD and DPSGD as the baseline algorithms. The results are summarized in Figure 10. From the figure, we can find that the proposed algorithm approximates the accuracy of CPSGD algorithm and outperforms the DPSGD algorithm. The average accuracy of the CPSGD algorithm decreases slightly in the later rounds. It is because that the weight divergence increases as the iteration proceeds due to non-iid data distribution [36]. Our proposed algorithm runs fewer rounds when achieving the same performance as the DPSGD algorithm, which reduces the computational complexity. The reasons are as follows. Under the non-iid setting, data distribution in the neighborhood cannot reflect the data distribution across the network. Thus, if models are only aggregated in the neighborhood, they can only learn the local data features and therefore exhibit a bad performance on the test sets. However, since our algorithm utilizes the GNN to aggregate the model parameters across the network, the model on each device can actually learn the global data features. Besides, the impacts caused by the errors of GNN reaching average consensus is mitigated by local model updates. Thus, the proposed algorithm performs better than the DPSGD algorithm.

## 6. Conclusions and Future Directions

In this paper, we mainly investigate a decentralized learning architecture, which avoids the possible congestion to the central server in the centralized architecture. We propose a new decentralized learning scheme utilizing GNN aggregation for training generalized models in networks that can be modeled as undirected or balanced directed graphs. First, the GNN structure has been investigated to reach average consensus. Then, the training strategy for GNN has been designed to implement the scheme into practical use. Furthermore, we introduce how to implement the GNN aggregation in a decentralized manner. Finally, simulations results demonstrate that the proposed algorithm is able to converge to near-optimal results owing to the global aggregation by GNN. Besides, benefiting from the multi-layer structure and nonlinear function of GNN, it is robust to the link loss as well as partial device participation. This initial study suggests the great potential of GNN being used for decentralized learning. Further research directions include considering the communication compressing scheme as well as extension to different network topologies. In addition, improving the training of GNNs and ameliorating the model architecture are promising means to fine-tune our proposal.

## Figures and Tables

**Table 1:** Comparisons Between Different Decentralized Learning Algorithms.

Reference	Methods	Description	Advantages	Disadvantages
[6,7,8]	DGD	Update the optimization variables based on full gradients.	For strongly convex loss function, it converges to the optimum with diminishing step sizes.	It cannot guarantee the convergence to the optimum with constant step size and is time-consuming based on full gradients.
[9]	EXTRA	Use the gradient tracking technique for invariant network scenario and undirected graphs.	They guarantee the convergence to the optimum with constant step size for strongly convex loss function.	They consume a lot of time based on full gradients.
[10]	DIGing	Use the gradient tracking technique considering variant network scenario and directed graphs.		
[11]	DADMM	Decompose the initial problem into several sub-problems.	It establishes the linear convergence for strongly convex local objectives.	1. It cannot flexibly accommodate the communication- computation tradeoff 2. It requires additional hyperparameters to tune.
[12]	COLA	Use techniques from primal-dual optimization based on CoCoA.	It achieves communication efficiency while maintaining resilient to the changes in the data and network topology.	It can only be applied to linear models.
[13,14,15,16,17]	DPSGD	Train the models based on stochastic gradients and then uses doubly stochastic mixing matrix to reach consensus.	It can efficiently train most machine learning models based on stochastic gradients.	It only aggregates the model in the neighborhood instead of the global network. It would cause poor model performance under the non-iid setting.

**Table 2:** Abbreviations.

Abbreviation	Description
DPSGD	Distributed parallel stochastic gradient descent
CPSGD	Centralized parallel stochastic gradient descent
SGD	Stochastic gradient descent
GNN	Gragh neural network
RGGs	Random geometric graphs
iid	Independent and identically distributed
non-iid	Non-independent and identically distributed
MSE	Mean squared error
UAV	Unmanned aerial vehicle

**Table 3:** Comparisons Between Different Methods for MNIST/Fashion-MNIST Image Classification.

Method	Sampling Strategies	Filter Order	Accuracy	
			MNIST	Fashion-MNIST
CPSGD	/	/	92.25%	84.64%
DPSGD	/	/	92.22%	84.17%
Proposed Algorithm	Head-sampling	1	89.75%	76.99%
		20	90.21%	78.79%
	Uniform-sampling	1	88.89%	76.67%
		20	89.32%	78.62%
	Tail-sampling	1	88.17%	76.58%
		20	89.03%	77.57%

## Data Availability

MNIST dataset was analyzed in this study. This data can be found here: http://yann.lecun.com/exdb/mnist/ (accessed date: 10 September 2021).
